# A Case of Abscessing Ileocecal Monomorphic Epitheliotropic Intestinal T-cell Lymphoma

**DOI:** 10.7759/cureus.72169

**Published:** 2024-10-22

**Authors:** Anastasia I Bekyarova, Ina Kobakova, Snejana Spasova

**Affiliations:** 1 General and Clinical Pathology, Forensic Medicine and Deontology, Medical University of Varna, Varna, BGR; 2 General and Clinical Pathology, Forensic Medicine and Deontology, Dr. Marko Markov Specialized Hospital for Treatment of Oncological Diseases, Varna, BGR

**Keywords:** abdominal abscess, caecum, ileocaecal region, intestinal t-cell lymphoma, meitl, perforation

## Abstract

Monomorphic epitheliotropic intestinal T-cell lymphoma (MEITL), previously referred to as enteropathy-associated T-cell lymphoma (EATL) type II, is a rare type of intestinal extranodal T-cell lymphoma that arises from intraepithelial T-lymphocytes of the intestinal mucosa. Here, we report a case of MEITL with an unusual localization in the ileocecal region complicated by an adjacent abscess and perforation of colon ascendens in a 65-year-old male. The patient was admitted to the hospital with acute abdominal pain. A computed tomography (CT) scan revealed a circumferential cecal wall thickening. Following an initial period of improvement, the patient's condition deteriorated due to bowel perforation and inflammatory processes in the abdominal cavity. A right hemicolectomy and a latero-lateral ileo-transverse anastomosis were performed, and the subsequent histological examination revealed a population of monomorphic lymphoid blast cells with hyperchromatic nuclei, coarse chromatin, and irregular nuclear outline, forming cellular aggregates and infiltrating the intestinal wall from the submucosa to the serosa. The tumor cells were small to medium in size and demonstrated marked epitheliotropism. Immunohistochemistry (IHC) showed intense cytoplasmic CD45, membrane-cytoplasmic CD3, cytoplasmic Bcl-2, CD8, and membrane CD56 expression. The proliferation index Ki-67 was evaluated as high, being positive in more than 70% of the tumor cells. The patient died 39 days after the initial onset of symptoms.

## Introduction

Monomorphic epitheliotropic intestinal T-cell lymphoma (MEITL) is a rare type of intestinal extranodal T-cell lymphoma that arises from intraepithelial T-lymphocytes of the intestinal mucosa. It was previously referred to as enteropathy-associated T-cell lymphoma (EATL) type II. Currently, this neoplasm is classified as a distinct entity due to the absence of evidence supporting a coeliac disease-related etiology [1]. It is more prevalent in Asian and Hispanic populations and predominantly affects males in their fifth to seventh decades of life [2]. The symptoms of MEITL are often nonspecific, including abdominal pain, distension, chronic diarrhea, unintentional weight loss, and fever. Bowel obstruction, perforation, and peritonitis are common complications of MEITL that require emergency surgery [3,4]. The most frequently affected site is the jejunum, followed by the ileum [3]. However, cases with multifocal lesions have also been reported in the literature [5]. Genetic studies have identified associations between MEITL and mutations in several genes, including SETD2, STAT5B, JAK3, TP53, JAK1, BCOR, and ATM. Additionally, defective H3K36 trimethylation has been observed in some cases. The disease course is aggressive, with a median overall survival (OS) of less than one year [6]. The rarity of the neoplasm, the low OS, and the resulting absence of large clinical studies present significant challenges regarding disease management and treatment strategies [3].

The aim of this study is to report a case of MEITL with an unusual localization in the ileocecal region, which was complicated by an abscess and perforation of the colon ascendens.

## Case presentation

**Figure 1:**
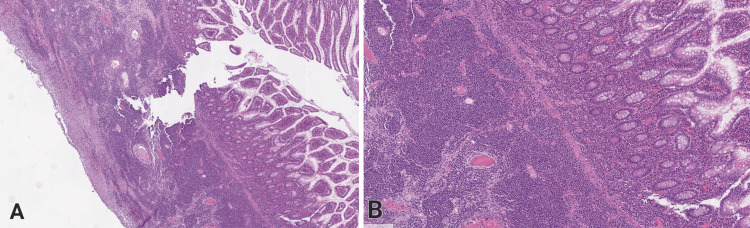
Histological appearance of MEITL, which is infiltrating the intestinal wall (cecum), H&E (A) Low magnification, 100×. (B) Higher magnification, 200×. H&E: hematoxylin and eosin; MEITL, monomorphic epitheliotropic intestinal T-cell lymphoma

We present a case of a 65-year-old male who was admitted to the hospital with acute abdominal pain. Clinical laboratory tests found evidence of iron deficiency anemia. The patient had no history or clinical signs of celiac disease. Symptomatic treatment with spasmolytics and analgesics was initiated. An abdominal computed tomography (CT) scan revealed a circumferential thickening of the wall of the cecum and the proximal part of the colon ascendens, with a thickness of up to 12 mm. The CT scan raised suspicion of tumor formation in the ileocecal region, and the patient was referred for further positron emission tomography (PET/CT) after discharge from the hospital. A diagnostic colonoscopy was conducted, yet no signs of an exophytic neoplastic process within the thickened intestinal region were identified. After initial improvement and temporary symptom resolution, the patient was released from the hospital. Two days later, he experienced rapid deterioration with pain in the right lower abdominal quadrant, distension, and profuse vomiting. Readmission and operative treatment were required due to bowel perforation. Intraoperatively, the surgical team found extensive fibrinous-purulent peritonitis with whitish serous plaques and an abscess cavity, about 5 cm in diameter, perforating the intestinal wall in the ileocecal region. A right hemicolectomy and a latero-lateral ileo-transverse anastomosis were performed. Necroinflammatory changes were observed in the cecum, colon ascendens, peritoneum, and retroperitoneum. In addition, the frozen section procedure showed abundant lymphoid infiltration within the resected ileocecal segment. After fixation of the specimen, a population of monomorphic lymphoid blast cells with hyperchromatic nuclei, coarse chromatin, and irregular nuclear outline was described, forming cellular aggregates and infiltrating the intestinal wall from the submucosa to the serosa (Figure 1A and Figure 1B).

**Figure 2:**
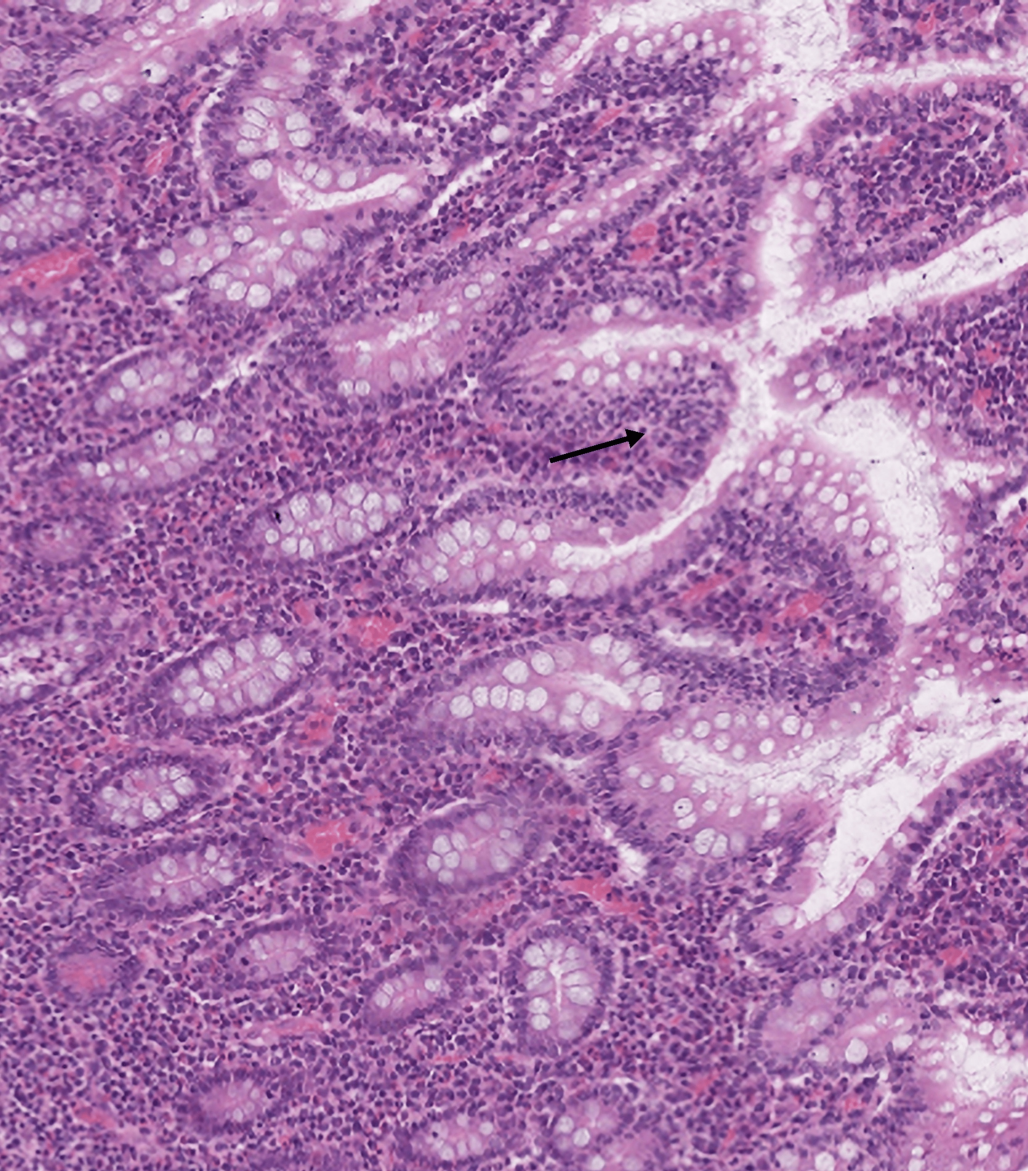
Monomorphic lymphoid blast cells showing marked epitheliotropism (arrow), 300×, H&E H&E: hematoxylin and eosin

The tumor cells were small to medium in size and demonstrated marked epitheliotropism (Figure 2).

**Figure 3:**
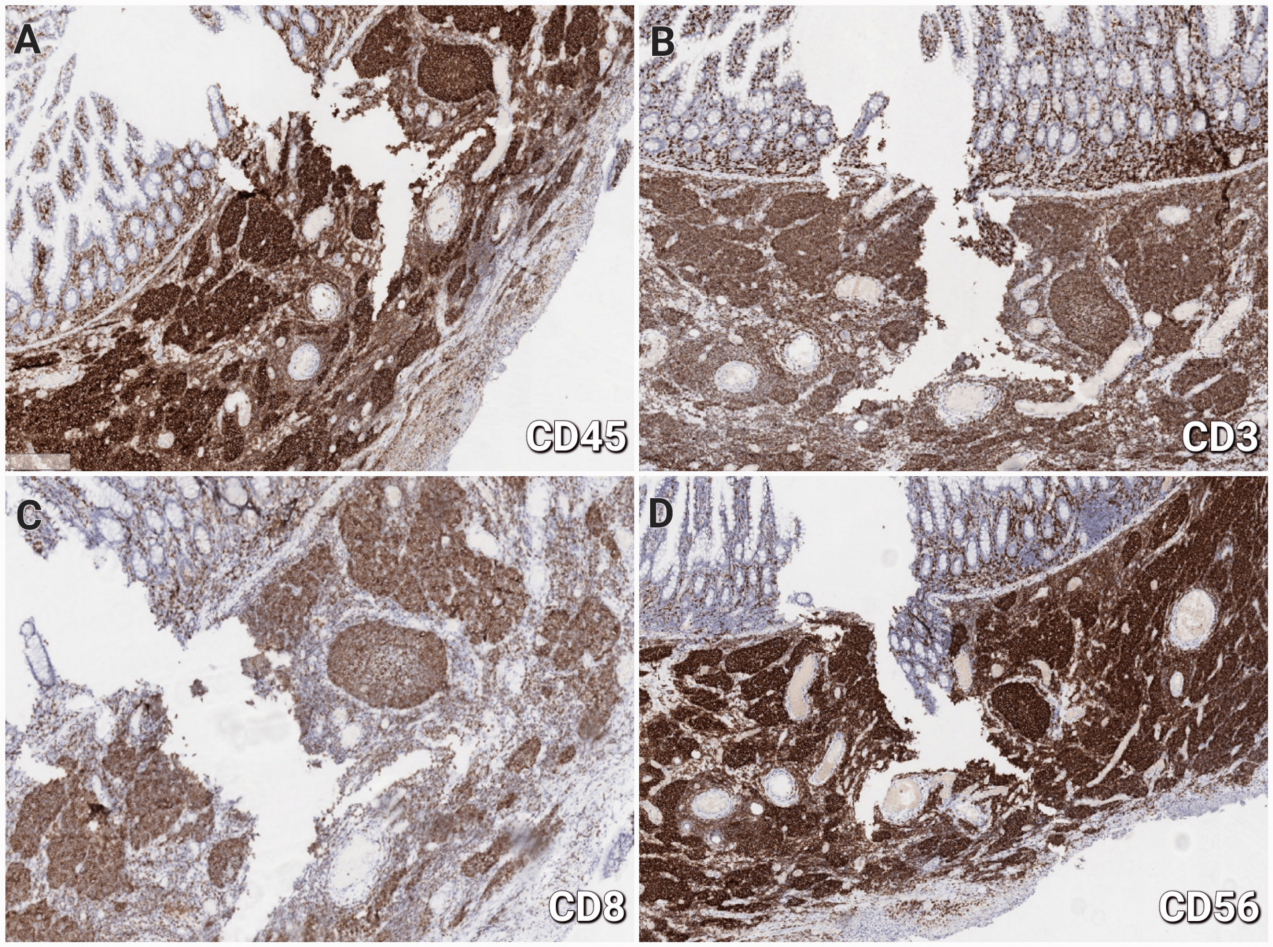
Immunohistochemical analysis of the lesion demonstrating expression of CD45 (A), CD3 (B), CD8 (C), and CD56 (D) in the tumor tissue, 200×

Immunohistochemistry (IHC) was required to ascertain the precise diagnosis, and a panel of IHC markers comprising CD3, CD4, CD5, CD8, CD10, CD15, CD20, CD23, CD30, CD45, CD56, Bcl-2, Bcl-6, Cyclin D1, and Ki-67 was performed. The tumor cells had intense cytoplasmic CD45 (Figure 3A), membrane-cytoplasmic CD3 (Figure 3B), cytoplasmic Bcl-2, CD8 (Figure 3C), and membrane CD56 expression (Figure 3D). CD45 indicates the lymphoid origin of the neoplasm, CD3 and CD8 are usually expressed in T-cell lymphomas, Bcl-2 expression is characteristic of extranodal lymphomas, and CD56 is an NK-cell molecular marker and is associated with epitheliotropism.

**Figure 4:**
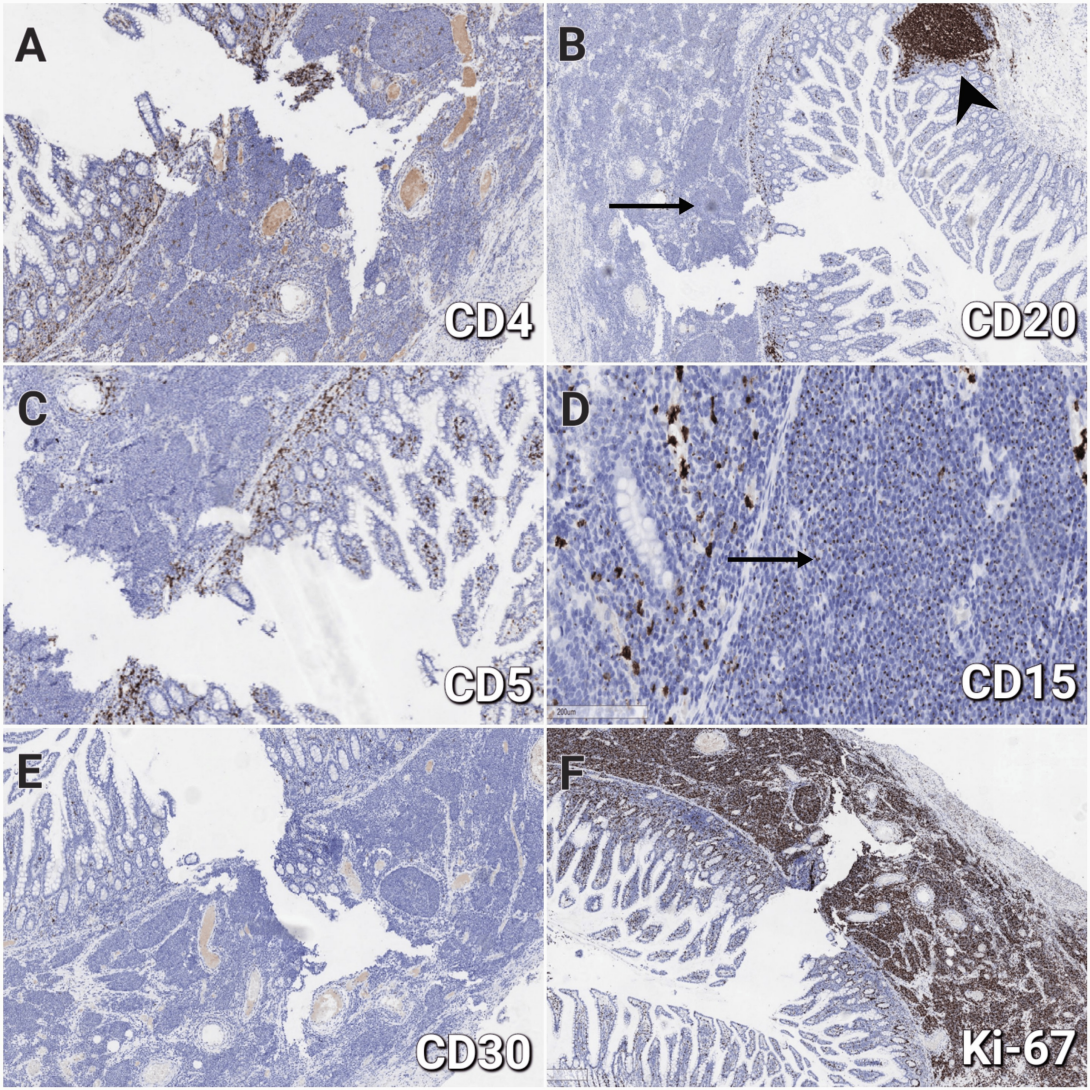
Immunohistochemical examination of the tumor (A) Focal expression of CD4 in 20% of the tumor cells, 200×. (B) Negative reaction for CD20 in the tumor cells (arrow) and positive in a small aggregate of B-cells (arrowhead), 100×. (C) Negative reaction for CD5, 200×. (D) Negative reaction for CD15 with a discreet positivity in intracytoplasmic organelles (arrow), 400×. (E) Negative reaction for CD30, 200×. (F) High Ki-67 expression (>70%) within the neoplasm, 100×. CD: cluster of differentiation; Ki-67: proliferation index

Focal membrane expression of CD4 (Figure 4A) was registered in approximately 20% of the tumor cells, interpreted as diagnostically insignificant. Negative IHC reaction was reported for CD20 (Figure 4B), CD23, CD5 (Figure 4C), CD10, Bcl-6, and Cyclin D1, excluding B-cell origin of the neoplasm. Negative CD15 (Figure 4D) and CD30 (Figure 4E) samples ruled out Hodgkin lymphoma as a potential diagnosis. CD15 showed positivity in intracytoplasmic organelles, which was marked as nonspecific for the diagnosis in the particular case. The proliferation index Ki-67 was evaluated as high, being positive in more than 70% of the tumor cells (Figure 4F). Based on the specific localization, the histology, and the IHC results, the tumor was determined as a MEITL.

The patient died 39 days after the initial onset of symptoms with signs of acute left-sided heart failure and acute renal failure. This occurred prior to the availability of the final results of the IHC analysis.

## Discussion

Extranodal non-Hodgkin lymphomas are frequently observed in the gastrointestinal tract (GIT), but their occurrence in the large intestine is exceedingly uncommon, accounting for only 10% of the cases. Moreover, primary colorectal lymphomas comprise less than 1% of all colorectal malignancies [7]. The majority of these lymphoid neoplasms originate from B-cells, whereas T-cell subtypes have a markedly lower incidence in Western populations [8]. In all cases, the diagnosis is based on detailed pathomorphological observation and the application of a specific combination of immunohistochemical antibodies [1]. The differential diagnosis of MEITL may include EATL, extranodal natural killer/T-cell lymphoma, indolent T-cell lymphoma of GIT, indolent natural killer cell lymphoproliferative disorder of GIT, intestinal T-cell lymphoma not otherwise specified, and peripheral T-cell lymphoma secondarily involving the GIT [9].

MEITL has been predominantly documented in the jejunum and the ileum [3]. However, cases with unusual localization have been described in the literature. Muramoto et al. reported a case of MEITL involving the stomach as the primary organ. The tumor presented with epigastric pain and irregular ulcers in the lesser curvature of the stomach on upper GIT endoscopy, subsequently complicated by a large gastric perforation [10]. Duodenal lesions are rare and may manifest with melena, along with obstruction and perforation [11,12]. In the colon, MEITL has been observed on a CT scan as a thickening of the intestinal wall, accompanied by the presence of multiple small ulcers in the edematous mucosa as endoscopic findings [13]. Secondary involvement of the bone marrow as a consequence of a disseminated process with an origin in the ileocecal region was reported by Zhang et al. [14]. Metastatic foci in the lung and brain were documented in a patient with a primary small intestine MEITL, which was complicated by a pelvic abscess and panperitonitis [15]. In our case, there is a possibility that the tumor arose from the cecum and subsequently invaded the surrounding tissue, ultimately reaching the ileocecal valve. The formation of an abscess may be closely related to the ability of the tumor to cause perforations, thus allowing contamination of surrounding tissues with intestinal microflora, which can result in acute purulent inflammation.

This type of lymphoma is known for its aggressive nature, late onset of symptoms, rapid progression, and poor response to conventional therapy [2,3]. Surgical resection as a definitive treatment is ineffective, offering minimal benefit in terms of life expectancy [6]. Despite the administration of CHOP (cyclophosphamide, doxorubicin, vincristine, and prednisone) and other CHOP-based therapies, such as CHOEP (CHOP plus etoposide) and Ro-CHOP (CHOP plus romidepsin), the tumor often remains unaffected due to chemoresistance [6]. Radiation therapy has been performed as a palliative care alternative to chemotherapy for patients with a higher risk of bowel perforation, achieving satisfactory results [16]. However, the median survival is reported to be approximately seven and a half months after confirmation of the diagnosis [2]. The prognosis is even more unfavorable in cases with bowel perforation, reaching only four months median OS, due to delayed initiation of chemotherapy and rapid worsening of the general status [4].

## Conclusions

In conclusion, MEITL is an aggressive neoplasm with an insidious clinical course, typically presenting at a late stage with associated complications. The primary localization is not exclusively in the small intestine and thickening of the gastrointestinal wall should be suggestive for MEITL elsewhere in the GIT. Current therapeutic strategies are rarely able to significantly improve survival or induce remission, underscoring the need for further research into new treatment options.
